# Antimicrobial Resistance, Biofilm Formation and *mec*A Characterization of Methicillin-Susceptible *S. aureus* and Non-*S. aureus* of Beef Meat Origin in Egypt

**DOI:** 10.3389/fmicb.2016.00222

**Published:** 2016-02-29

**Authors:** Kamelia M. Osman, Aziza M. Amer, Jihan M. Badr, Nashwa M. Helmy, Rehab A. Elhelw, Ahmed Orabi, Magdy Bakry, Aalaa S. A. Saad

**Affiliations:** ^1^Department of Microbiology, Faculty of Veterinary Medicine, Cairo UniversityGiza, Egypt; ^2^Department of Pharmacology, Faculty of Veterinary Medicine, Cairo UniversityGiza, Egypt; ^3^Department of Poultry Diseases, Animal Health Research InstituteCairo, Egypt; ^4^Departments of Biotechnology, Animal Health Research InstituteGiza, Egypt; ^5^Department of Microbiology, National Research CenterCairo, Egypt

**Keywords:** methicillin- resistant-susceptible *S. aureus*, methicillin- resistant-susceptible non-*S. aureus*, beef meat, antibiotic resistance genes, biofilm

## Abstract

Methicillin-resistant *Staphylococcus aureus* (MRSA) have been found in various farm animal species throughout the world. Yet, methicillin-susceptible *S. aureus* (MSSA), methicillin-susceptible non-*S. aureus* (MS-NSA), and methicillin-resistant non-*S. aureus* (MR-NSA) were not investigated. Therefore, we persued to determine the diversity in their phenotypic virulence assay, phenotypic antimicrobial resistance profile and molecular characterization in one of the food chains in Egypt. Samples were collected during 2013 from beef meat at retail. Twenty seven isolates comprising five species (*S. hyicus, S. aureus, S. schleiferi* subsp*. coagulans, S. intermedius*, and *S. lentus*) were characterized for their antibiotic resistance phenotypic profile and antibiotic resistance genes (*mec*A, *cfr, gyrA, gyr*B, and *grlA*). Out of the 27 *Staphylococcus* isolates only one isolate was resistant to the 12 antibiotics representing nine classes. Raw beef meat sold across the Great Cairo zone, contains 66.7% of MRS, with highest prevalence was reported in *S. aureus* (66.7%), while the MRS non-*S. aureus* strains constituted 66.7% from which *S. hyicus* (60%), *S. intermedius* (33.3%), *S. schleiferi* subsp. *coagulans* (100%), and *S. lentus* (100%) were MRS. Seven *S. aureus*, six *S. hyicus*, four *S. schleiferi* subsp. *coagulans*, three *S. intermedius*, and one *S. lentus* isolates although being resistant to oxacillin yet, 11/27 (40.7%) carried the *mec*A gene. At the same time, the *cfr* gene was present in 2 of the nine *S. aureus* isolates, and totally undetectable in *S. hyicus, S. schleiferi* subsp. *coagulans, S. intermedius*, and *S. lentus.* Although, global researches largely focused into MRSA and MR-NSA in animals on pigs, the analysis of our results stipulates, that buffaloes and cattle could be MRSA dispersers and that this theme is not specific to pigs. Detection of MSSA virulence determinants is a must, as although oxacillin resistance may be absent yet, the MSSA may carry the virulence determinants which could be a source of perilous *S. aureus* for the human community.

## Introduction

In Egypt, since more than three millennia dating from the Eighteenth Dynasty chronology, the domestic cattle are incorporated with all types of agricultural activities in addition to their provision of meat, internal organs (heart, kidneys, pancreas, rumen, intestine), milk, cheese, ghee, skin, bone, manure, horns, and tendons (Conférence publique de Mlle M-Christine Lavier Toulon, 2000; Benderitter, 2003).

Antimicrobial agents are widely spread and intensively used in the food production systems (FAO, 2015). The aftermath of this operation varies considerably globally as a result of the interaction between human (social structure) and animal demography (species, distribution, and density), farming systems, contaminated water sources, socio-economic strategies (manufacturing, commerce, availability of food, and animal fitness), and nation and world-wide trade. Although, antibiotic use in food animals has been concealed from the public scene yet, antibiotic application in meat producing animals have impacted on public health and has left no room for confusion after being scientifically documented (Conniff, 2015). In 2010, an announcement having a public *consequential impact* was made public by the FDA to the world in that 28.7 million pounds of antibiotics, which comprises 80% of the total amount of antibiotics utilized in the USA, were being poured down in the animal production industry not to medical care and that currently, two million people are annually sickened by resistant bacterial infections and that 23,000 die annually according to the CDC (Conniff, 2015).

Eventually, besides tracing the use of antibiotics, the effectual monitoring of antimicrobial resistance is the linchpin of any national and international successful achievement in their control policy. The pivotal role of monitoring was highlighted in the 2001 WHO Global Strategy for Containment of antimicrobial resistance and on the occasion of World Health Day 2011, wherein resistance, antimicrobial consumption, and disease stress are included as prime listings. The facts and statistics collected can be used to implement the government's rationale for introducing such radical legislation in the perfection of antibiotic use, to propose strategy and discern the prime concern for employment to provide public support regionally and globally.

*Staphylococcus aureus* is a hazard because of its detrimental effects on animal health and its capacity for transmission from animals to humans and vice-versa (Peton and Le Loir, 2014). The isolation universality of multidrug-resistant staphylococci from meat samples has been perceived by several researchers (Santos-Sanches et al., 2000; Khan et al., 2005; Huber et al., 2011; Bhargava and Zhang, 2014; Chajęcka-Wierzchowska et al., 2015; Guran and Kahya, 2015). This should be taken seriously, especially when the finding of Waters et al. (2011) documents that, close to 50 percent of meat samples tested from U.S. grocery stores are contaminated with the bacteria *S. aureus* and resistant to at least three classes of antibiotics. Methicillin-resistant strains of *S. aureus* (MRSA), which is linked to a wide range of human diseases (Köck et al., 2010; Fätkenheuer and Kaasch, 2014; Yaw et al., 2014), is notorious for its related-Nous to food contamination and its potentiality for its transmission to humans prevails. The pre-eminent disquiet in the European Union, has become the prevention and control of MRSA being ascertained as the most public health problem (Köck et al., 2010), an act that should be implemented in Egypt. Also, the high prevalence of MR-CNS found in livestock production (Huber et al., 2011) becomes a relevant consternation in view of the dispersing potentiality of *mec*A to MRSA as several coagulase negative staphylococcus (CNS) species, which are also non-*S. aureus* (NSA), can serve as reservoirs for *mec*A (Bhargava and Zhang, 2014). This consigned multiresistant CNS-NSA strains the intuitive capability to develop into an emerging problem for veterinary medicine.

Although CNS have emerged as important pathogens, little is known about the virulence factors of these bacteria (Stepanovic et al., 2001). The most important virulence factor of CNS is assumed to be the capacity for biofilm formation and, that testing for biofilm production could be a useful marker for the pathogenicity of CNS (Stepanovic et al., 2001).

In general, we have not come across any investigations involving biofilm formation, and the antibiotic resistant genes *mec*A, *cfr, gyr*A, *gyr*B, and *grl*A in MRSA in beef meat and in methicillin-resistant non-*S. aureus* (MR-NSA). Thereby, we aimed an endeavor to identify one of the main hazardous threat to public health, *Staphylococcus* species, to determine the generality of contamination of beef meat as a measurement for the exposure of consumers to MRSA and MR-NSA in retail beef meat in the Egyptian market, the rate of biofilm producers among the tested *S. aureus* comparable to non-*S. aureus strains*, their carriage to the antibiotic resistant genes *mec*A, *cfr, gyr*A, *gyr*B, and *grl*A and their consequence as a public health hazard.

## Materials and methods

### Sample collection

During the year 2013, 100 samples of retail whole beef were collected from several butchers and marts in the Great Cairo zone. Each sample weighing at least 500 g was aseptically collected with the minimum of cross-contamination and immediately conveyed to the laboratory to be microbiologically examined.

### Isolation and identification

To 50 g of meat samples, 100 ml of phosphate-buffered saline was added all in a sterile plastic bag which were vigorously shaken for 2 min to remove bacteria. Then 1 ml of each rinsate was used for isolation and detection of *Staphylococcus* isolates by the Double Enrichment method. The isolates were used for further culture to be consequently biochemically characterized as *S. aureus* (*n* = nine) and non-*S. aureus* (*n* = 18) according to Thorberg and Brandstrom (2000). A sub-culture of the 27 isolates were stored at −27°C in tryptic soy broth and fetal calf serum [Gibco/Invitrogen Carlsbad (CA) USA] containing 2% yeast extract (v/v) to be revitalized for phenotypic virulence assay, phenotypic antimicrobial resistance profile and molecular characterization.

### Phenotypic virulence assays

For the 27 isolated staphylococci, four virulence factors were evaluated (alpha, beta and delta hemolysins, cytotoxicity, and biofilm formation) using phenotypic tests.

### Hemolysis pattern on blood agar

The 27 tested isolates were subcultured onto 5% defibrinated sheep and rabbit blood (Merck, Darmstad, Germany) at 37°C for 24 h according to the procedure of Futagawa-Saito et al. (2006) to obtain isolated colonies. The type of hemolysis was recorded as α-, β-, and double (α + β). After incubation at 37°C for 24 h the clear zone around the colonies was registered as positive reaction. Some *S. aureus* strains proved the presence of CAMP-like factor, a special hemolysin, the activity of which is revealed by streaking the bacterial studied strain on 5% sheep blood agar, at 8 mm distance perpendicularly on the β-hemolytic *S. aureus* ATCC 25923 reference strain, and incubating the plate aerobically at 37°C for 24 h. The occurrence of synergic β-haemolysis at the confluence of the two spot areas indicates the presence of CAMP-like factor. The *S. aureus* (ATCC 25923 and ATCC 49775) and *S. intermedius* Hajek (ATCC strain 49052) were used as reference strains.

### Cytotoxicity assay

The cytotoxicity assay was carried out using Vero (African green monkey kidney) cells in 96-well microtiter trays according to the protocol of Tao et al. (1999). Suspensions of each of the 27 staphylococcal strains in distilled water were adjusted to 0.5 McFarland standard. Then, 20 μl of bacterial suspensions were added to 3.5 ml of brain heart infusion broth (BBL, Becton Dickinson Microbiology Systems). The tubes were incubated 2 days at 35°C, and thereafter for 2 days at room temperature. After centrifugation of bacterial broth cultures, 20 μl of supernatants were added in triplicate to 180 μl of cell culture medium. After incubation at 37°C, in a humid atmosphere and 5% CO_2_, the cytotoxic effect of each staphylococcal strain on the Vero monolayer morphology was microscopically perceived up to 5 days.

### Biofilm formation

Biofilm formation assay was determined by the Christensen's tube method and the Congo red agar assay (CRA) as proposed by Osman et al. (2015, 2016). The biofilm-producer ATCC 25923 and the biofilm negative ATCC 12228 were used as reference strains.

### Phenotypic characterization of slime production in CRA

This method is based on the characteristic cultural morphology of biofilm-forming bacteria on Congo red medium. The strains were cultured on CRA plates, composed of brain heart infusion broth (BHI) (Oxoid Ltd, Hampshire, England) 37 g/l, 10 g/l Congo red and 50 g/l sucrose and agar No 1 (Oxoid Ltd, Hampshire, England) 10 g/l. The plates were subsequently incubated for 24 h at 37°C and overnight at room temperature. The production of black colonies with a dry crystalline consistency by the organisms was taken to indicate slime production as non-slime-producing strains develop red colonies. ATCC 35982, a non-slime-producing strain (−), ATCC 35983, a moderate slime producing strain (++), and ATCC 35984, a strong slime producing strain (++), served as controls.

### Investigation of biofilm production by adherence to borosilicate test tubes

A qualitative assessment of biofilm formation was determined by tube assay. An overnight culture of each of the 27 isolated staphylococci were grown in MHB and incubated for 24 h at 37°C. The tubes were removed and washed with phosphate buffer saline and dried. Tubes were stained with 0.1% crystal violet for 10 min. Excess stain was removed and tubes were washed with distilled water. Tubes were then dried and observed for biofilm formation. Biofilm formation was considered positive when a visible film lined the wall and bottom of the tube. Ring formation at the liquid-air interface was not indicative of biofilm formation. The scoring for tube method was done according to the results of the control strains. The isolates were classified into three categories: Biofilm formation was considered positive when a visible film lined the wall and the bottom of the tube. The amount of biofilm formed was scored as 1-weak/none, 2-moderate, and 3-high/strong.

### Phenotypic antibiotic resistance profile

Resistance of the 27 isolated staphylococci to 12 specific antibiotics (**Table 3**) were validated by culturing each isolate on *Mueller-Hinton agar (MHA)* supplemented with 0.5% NaCl and 2% for oxacillin, to obtain a fresh culture, to be determined by the disk diffusion method according to procedures of the CLSI (2012). Antimicrobials on the WHO's critically important antimicrobials list (2012) were selected for testing according to their importance to human and animal health (FAO/WHO/OIE, 2008; WHO, 2009). One loopful of colony material was subcultured in 5 ml Mueller-Hinton broth for an overnight at 35°C, vortexed well and adjusted to obtain a turbidity comparable to that of 0.5 McFarland opacity standard (bioMe'rieux, Marcy 1'Etoile, France). The bacterial suspensions were streaked on MHA plates with a cotton swab and with an antibiotic disc dispenser, the discs were placed on the agar surface, plates were incubated at 37°C, the standard temperature used for antimicrobial susceptibility testing, for 24 h, and the diameter of the inhibition zones was measured. The isolates were classified as sensitive, intermediate and resistant based on the diameter of the clearing zone according to CLSI (2012) guidelines. The antibiotic discs represented nine classes of antibiotics: ampicillin-sulbactam, methicillin, penicillin, and oxacillin (Penicillins); chloramphenicol (Chloramphenicols); ciprofloxacin (Florinated quinolones); clindamycin (Lincosamides); gentamycin (Aminoglycosides); erythromycine (Macrolides); sulphamethoxazole/trimethoprim (Sulphonamides); tetracycline (Tetracyclines); vancomycine (Glycopeptides). *S. aureus* ATCC 29213 (β-lactamase positive), and *S. aureus* ATCC 25923 (β-lactamase negative) were used as quality control. Multidrug resistance was reported as a single isolate resistant (intermediate or complete) to 3 or more antimicrobial classes (Waters et al., 2011). Bacteria strains that were resistant and intermediate were categorized as non- susceptible while the sensitive strains were categorized as susceptible.

### DNA extraction

Bacterial DNA from the 27 staphylococci were isolated from an overnight cultures using the boiling protocol previously described (Sowmya et al., 2012). DNA precipitates were resuspended in an appropriate volume of Tris-EDTA buffer solution.

### PCR for detection of genes encoding antimicrobial resistance

The 27 *Staphylococcus* species isolates were confirmed as MSSA, MRSA, methicillin-susceptible non-*Staphylococcus aureus* (non-MSSA), and methicillin-resistant non-*Staphylococcus aureus* (non-MRSA) molecularly to identify *mec*A (the gold-standard reference method for this analysis). In addition, four antimicrobial resistance genes responsible for quinolone resistance (*gyrA, gyr*B, and *grlA* genes) which are habitually found in *S. aureus* according resistance in the quinolone-resistance-determining-regions (QRDRs) and responsible for quinolone resistance (*gyrA, gyr*B, and *grlA* genes) and the *cfr* gene which confers resistance to five antibiotic classes (oxazolidinones, phenicols, streptogramin compounds, lincosamidins, and pleuromutilins usually referred to as PhLOPSA phenotype) were also amplified. Annealing temperatures and oligonucleotides used are presented in Table 1. The PCR runs included a reagent positive and negative control (without template DNA). Three independent PCR amplifications were carried out with a GeneAmp PCR System 2400 (Perkin-Elmer, Weiterstadt, Germany). Two microliters of template DNA was added to 23 mL of distilled sterile water, and finally 25 mL of reaction mixture containing 10mM Tris-HCl (pH8.3), 50mM KCl, 2.5mM MgCl_2_, 100 mM deoxynucleoside triphosphates, 1 mM of each primer and 3U of Taq polymerase were added. The amplicons were analyzed by electrophoresis on 1.5% (w/v) agarose gel in TBE buffer.

**Table 1:** **Antimicrobial resistance genes targets, primers and nucleotide sequences**.

**Gene designation**	**Oligonucleotide sequences (5′-3′)**	**Amplion size (bp)**	**PCR conditions**	**References**
*mecA*	**F:** GTAGAAATGACTGAACGTCCGATAA **R:** CCAATTCCACATTGTTTCGGTCTAA	**310**	Initial denaturation: 95°C for 1 min Amplification (30 cycles of) Denaturation: 95°C for 30 s Annealing: 50°C for 30 s Extension: 72°C for 60 s Final extension: 72°C for 4 min	Spanu et al., 2004
*gyrA*	**F:** ATGGCTGAATTACCTCAATC **R:**CATCATAGTTATCGATGAAATC	**399**	Initial denaturation: 95°C for 1 min Amplification (30 cycles of) Denaturation: 95°C for 30 s Annealing: 55°C for 30 s Extension: 72°C for 60 s Final extension: 72°C for 4 min	Dubin et al., 1999
*grlA*	**F:** AATACGTATGATAAGAATTTCCG **R:** GTTGTGTCATCATAGTTTGG	**429**		Dubin et al., 1999
*gyrB*	**F:** CAGCGTTAGATGTAGCAAGC **R:** CCGATTCCTGTACCAAATGC	**250**		Linde et al., 2001
*cfr*	**F:** TGAAGTATAAAGCAGGTTGGGAGTCA **R:** ACCATATAATTGACCACAAGCAGC	**746**	Initial denaturation: 94°C for 1 min Amplification (34 cycles of) Denaturation: 94°C for 1 min Annealing: 48°C for 30 s Extension: 72°C for 3 min Final extension: 72°C for 7 min	Kehrenberg and Schwarz, 2006

## Results

### Identification of staphylococci

We identified 27 staphylococcal isolates belonging to five different staphylococcal species. These included *S. hyicus* (*n* = 10, 37%), *S. aureus* (*n* = 9, 33.3%), *S. schleiferi* subsp. *coagulans* (*n* = 4, 14.8%), *S. intermedius* (*n* = 3, 11.1%), and *S. lentus* (*n* = 1, 3.7%).

### Phenotypic virulence assays

Summarized results of examining *the 27 staphylococci isolates* for factors that possibly contribute to their virulence are presented in Table 2.

**Table 2:** **Virulence factors assay of the *Staphylococcus* spp. Isolates**.

***Staphylococcus* spp**.	***n* = isolates**	**Hemolysis, Number of isolates showing**			**Cytotoxic assay^a^, Number of isolates showing**		**Assessment of Biofilm formation results**			
		**β**	**α**	**γ**	**Cell lysis**	**Cell rounding**	**CR, Number of isolates showing**		**CTM, Number of isolates showing**	
							**Slime +**	**Slime −**	**Positive for biofilm production**	**Negative for biofilm production**
*S. aureus*	9	6	2	1	9	0	4	5	9	0
*S. hyicus*	10	9	1	0	10	0	3	7	10	0
*S. intermedius*	3	2	0	1	3	0	1	2	3	0
*S. schleiferi* subsp. *coagulans*	4	3	1	0	4	0	3	1	2	2
*S. lentus*	1	0	0	1	1	0	0	1	0	1

a Bacteria were grown in brain heart infusion broth overnight and loaded onto Vero cell monolayers. After 2 days of incubation at 35°C in 95% air and 5% CO_2_, the cells were examined microscopically for morphological changes. Cell lysis was indicated by Vero cell destruction. Cell rounding indicated Vero cells detached from the plastic cultural surface and floating in the cell culture medium.

CRA, Congo red agar. Slime production of the 27 staphylococcal strains of beef meat origin.

CTM, Christensen's tube method.

### Haemolysin production

Haemolytic activity on plates with 5% defibrinated rabbit or sheep blood was established. In the strains showing hemolytic activity, the three patterns of haemolysis (β, α, and γ) were observed, 66.7% were demonstrated to be β-hemolytic, 22.2% were α-hemolytic, and 10.1% were γ-hemolytic.

### Cytotoxicity assay

All of the 27 *Staphylococcus* isolates were cytotoxic to Vero cells.

### Biofilm producing phenotype

We found a highly resistant pattern among biofilm producers in comparison with non-biofilm producers. Among the 7/9 vancomycin resistant *S. aureus* isolates, they were also a biofilm producer (77.8%). Generally, the 20 vancomycin resistant *S. aureus* and non-*S. aureus* species were also biofilm producers. The affinity of the 27 *Staphylococcus* species to form a biofilm was found to be in favor of the glass surface by 88.9% (24/27).

### Tube method

The qualitative tube adherence test depends on the visual assessment of the degree of adherence of *Staphylococcus* isolates to the sides of borosilicate test tubes. The 27 *Staphylococcus* isolates were screened for biofilm formation by tube method. Results of the screening for biofilm by the tube method is shown in Table 2. Out of the 27 *Staphylococcus* isolates, 24 (88.9%) were strongly positive to the ability of biofilm formation, while 3 (11.1%) were negative to biofilm formation. Biofilm formation affinity was observed in the nine *S. aureus* isolates (100%) and 15/18 in the non-*S. aureus* isolates (83.3%).

### Congo red agar method

Among the 27 *Staphylococcus* isolates, 11 (40.7 %) showed black colonies with dry crystalline consistency which is indicative of slime production whereas 16 (59.3 %) isolates showed pink colored colonies with mucoid appearance on the CRA plates which were taken as negative for slime production (Table 2). Ability to produce slime was evident in 4/9 (44.4%) of the *S. aureus* and 7/18 (38.9%) of the non-*S. aureus* isolates. The studies on slime formation by the non-*S. aureus* species of medical importance revealed that *S. hyicus, S. schleiferi* subsp. *Coagulans*, and *S. intermedius* showed that up to 30% (3/10), 75% (3/4) and 33.3% (1/3), respectively, of these species produced slime, while *S. lentus* (1/1) was unable to produce slime.

### Antibiotic resistance phenotypes for all staphylococci

The phenotypic resistance pattern, prevalence and diversity of the 27 *Staphylococcus* species isolated from the beef meat are recorded in Table 3. The 27 *Staphylococcus* species were resistant to the 12 antibiotics with variable degrees as to each of the antibiotics used as indicated in Table 3 to reveal in ascending order: ampicillin-sulbactam 14.8% (4/27), chloramphenicol 29.6% (8/27), erythromycin 48.1% (13/27), ciprofloxacin 59.3% (16/27), methicillin 63% (17/27), tetracycline 66.7% (18/27), vancomycine 74.1% (20/27), oxacillin 77.8% (21/27), penicillin 92.6% (25/27), clindamycin 96.3% (26/27), gentamycin 96.3% (26/27), and sulphamethoxazole/trimethoprim 100% (27/27). The different *Staphylococcus* species also reflect a difference in their antibiotic resistance to the 12 antibiotics each species with a different capacity to resistance: *S. lentus* 91.7% (11/12), *S. aureus* 77.8% (84/108), *S. intermedius* 72.2% (26/36), *S. schleiferi* subsp. *coagulans* 70.8% (34/48), and *S. hyicus* 55% (66/120).

**Table 3:** **Results of antimicrobial resistance tests by disk diffusion method for *Staphylococcus* species**.

**On the WHO's critically important antimicrobials list (2012)**	**Antibiotic disc**	**Concentration**	**Bacterial strains (Zone diameter values in mm)**				
			***S. aureus* (*n* = 9)**	***S. hyicus* (*n* = 10)**	***S. intermedius* (*n* = 3)**	***S. schleiferi* subsp. *coagulans* (*n* = 4)**	***S. lentus* (*n* = 1)**
**PENICILLINS**							
Critically important	Peniciilin	10 IU	20 (R, 9/9)	20 (R, 8/10)	20 (R, 3/3)	21 (R, 4/4)	21 (R, 1/1)
Critically important	Ampicillin-sulbactam	20 μg	14 (R, 2/9)	20 (R, 0/10)	21 (R, 0/3)	13 (R, 1/4)	15 (R, 1/1)
Critically important	Methicillin	5 μg	13 (R, 6/9)	14 (R, 5/10)	14 (R, 1/3)	13 (R, 4/4)	15 (R, 1/1)
Highly important	Oxacillin	1 μg	15 (R, 7/9)	17 (R, 6/10)	16 (R, 3/3)	16 (R, 4/4)	18 (R, 1/1)
**PHENICOLS**							
Highly important	Chloramphenicol	30 μg	10 (R, 4/9)	11 (R, 1/10)	11 (R, 1/3)	11 (R, 2/4)	21 (R, 0/1)
**FLUOROQUINOLONES**							
Critically important	Ciprofloxacin	5 μg	11 (R, 8/9)	12 (R, 4/10)	11 (R, 2/3)	12 (R, 1/4)	12 (R, 1/1)
**LINCOSAMIDES**							
Highly important	Clindamycin	2 μg	10 (R, 9/9)	10 (R, 9/10)	10 (R, 3/3)	10 (R, 4/4)	12 (R, 1/1)
**MACROLIDES**							
Critically important	Erythromycin	15 μg	10 (R, 7/9)	11 (R, 1/10)	11 (R, 2/3)	11 (R, 2/4)	12 (R, 1/1)
**AMINOGLYCOSIDES**							
Critically important	Gentamycin	10 μg	8 (R, 9/9)	9 (R, 9/10)	8 (R, 3/3)	8 (R, 4/4)	10 (R, 1/1)
**SULPHONAMIDES**							
Highly important	Sulfamethoxazole/Trimethoprim	25 μg	7 (R, 9/9)	7 (R, 10/10)	7 (R, 3/3)	8 (R, 4/4)	8 (R, 1/1)
**TETRACYCLINES**							
Highly important	Tetracycline	30 μg	10 (R, 7/9)	11 (R, 6/10)	11 (R, 2/3)	11 (R, 2/4)	10 (R, 1/1)
**GLYCOPEPTIDES**							
Critically important	Vancomycin	30 μg	10 (R, 7/9)	12 (R, 7/10)	10 (R, 3/3)	11 (R, 2/4)	10 (R, 1/1)

Zone diameter values. The results of the interpretation are indicated in brackets (R, Resistant). For disk zone diameter interpretation (CLSI, 2014) standards was used, (n =): number of isolates.

The several MDR combination patterns are recorded in Table 4. The Table reveals, that there were 21 different combination patterns ranging from four to twelve antibiotics in each combination. Out of the 27 *Staphylococcus* isolates only one isolate was resistant to the 12 antibiotics representing nine classes. This isolate was one of the nine *S. aureus* isolates (Table 5). The Table also reveals the wide dispersity in the number of antibiotic combinations in each isolate and species of the staphylococci isolates (*S. aureus, five* combinations; *S. hyicus*, five combinations; *S. intermedius*, three combinations; *S. schleiferi* subsp. *coagulans* two combinations; and *S. lentus, one* combination).

**Table 4:** **Multidrug resistance patterns among MRSA isolates from local beef retail meat**.

**Number of resistant Antibiotics**	**Antibiotics**	**Number of species**	**Total number of species (27)**	**%**
4	P, VA, SXT, CIP	1	1	3.5
6	P, CN, E, DA, VA, SXT	1	6	22
	CN, E, DA, TE, C, SXT	1		
	P, OX, CN, DA, VA, SXT	1		
	P, OX, MET, CN, DA, SXT	2		
	CN, DA, TE, VA, SXT, CIP	1		
7	P, CN, DA, TE, VA, SXT, CIP	1	4	15
	P, OX, CN, DA, VA, SXT, CIP	1		
	P, OX, MET, CN, DA, SXT, CIP	1		
	P, OX, MET, SAM, CN, DA, SXT	1		
8	P, OX, MET, CN, DA, TE, VA, SXT	1	4	15
	P, MET, CN, E, DA, TE, SXT, CIP	1		
	P, OX, CN, DA, TE, VA, SXT, CIP	2		
9	P, OX, CN, E, DA, TE, VA, SXT, CIP	1	4	15
	P, OX, MET, CN, DA, TE, VA, SXT, CIP	2		
	P, OX, MET, CN, E, DA, TE, SXT, CIP	1		
10	P, OX, MET, CN, E, DA, TE, VA, C, SXT	2	2	7
11	P, OX, MET, SAM, CN, E, DA, TE, VA, SXT, CIP	1	5	19
	P, OX, MET, CN, E, DA, TE, VA, C, SXT, CIP	3		
	P, OX, MET, SAM, CN, E, DA, VA, C, SXT,CIP	1		
12	P, OX, MET, SAM, CN, E, DA, TE, VA, C, SXT, CIP	1	1	3.5

C, Chloramphenicol; CIP, Ciprofloxacin; CN, Gentamycin; DA, Clindamycin; E, Erythromycin; MET, Methicillin; OX, Oxacillin; P, Peniciilin; SAM, Ampicillin-sulbactam; SXT, Sulfamethoxazole/Trimethoprim; TE, Tetracycline; VA, Vancomycin.

**Table 5:** **Antibiotic resistance phenotype and genotype patterns**.

***Staphylococcus* spp**.	**Resistance pattern**	**Number of antibiotics**	**Resistance Genes**				
			***mecA***	***gyrA***	***grlA***	***gyrB***	***cfr***
*S. aureus* (*n* = 9)	P, CN, E, DA, VA, SXT	6				+	
	P, OX, CN, DA, TE, DA, SXT, CIP	8		+	+		
	P, MET, CN, E, DA, TE, SXT, CIP	8	+		+		
	P, OX, CN, DA, TE, VA, SXT, CIP	8		+	+	+	
	P, OX, MET, CN, E, DA, TE, SXT, CIP	9		+	+		
	P, OX, MET, CN, E, DA, C, TE, VA, SXT, CIP	11	+	+	+		
	P, OX, MET, CN, E, DA, C, TE, VA, SXT, CIP	11	+	+	+		
	P, OX, MET, SAM, CN, E, DA, C, VA, SXT, CIP	11	+	+	+		+
	P, OX, MET, SAM, CN, E, DA, C, TE, VA, SXT, CIP	12	+	+	+		+
*S. hyicus* (*n* = 10)	P, VA, SXT, CIP	4		+	+		
	CN, E, DA, C, TE, SXT	6					
	P, OX, MET, CN, DA, SXT	6					
	P, OX, MET, CN, DA, SXT	6				+	
	CN, DA, TE, VA, SXT, CIP	6		+	+		
	P, CN, DA, TE, VA, SXT, CIP	7		+	+		
	P, OX, CN, DA, VA, SXT, CIP	7		+	+		
	P, OX, MET, CN, DA, TE, VA, SXT	8	+				
	P, OX, MET, CN, DA, TE, VA, SXT, CIP	9	+	+	+	+	
	P, OX, MET, CN, DA, TE, VA, SXT, CIP	9	+	+	+	+	
*S. intermedius* (*n* = 3)	P, OX, CN, DA, VA, SXT	6					
	P, OX, CN, E, DA, TE, VA, SXT, CIP	9		+	+	+	
	P, OX, MET, CN, E, DA, VA, SXT, C, TE, CIP	11	+	+	+		
*S. schleiferi* subsp. *Coagulans* (*n* = 4)	P, OX, MET, CN, DA, SXT, CIP	7		+	+		
	P, OX, MET, SAM, CN, DA, SXT	7			+	+	
	P, OX, MET, CN, E, DA, C, TE, VA, SXT	10	+				
	P, OX, MET, CN, E, DA, C, TE, VA, SXT	10	+				
*S. lentus* (*n* = 1)	P, OX, MET, SAM, CN, E, DA, TE, VA, SXT, CIP	11		+	+		
Total (*n* = 27)			11/27	17/27	19/27	7/27	2/27

C, Chloramphenicol; CIP, Ciprofloxacin; CN, Gentamycin; DA, Clindamycin; E, Erythromycin; MET, Methicillin; OX, Oxacillin; P, Peniciilin; SAM, Ampicillin-sulbactam;

SXT, Sulfamethoxazole/Trimethoprim; TE, Tetracycline; VA, Vancomycin.

### Widespread distribution of antibiotic resistance genes among staphylococcal isolates

We screened the meat *Staphylococcus* isolates for the presence of the multiple resistance genes *mec*A, *cfr, gyrA, gyr*B, and *grlA* genes among *Staphylococci* isolated from beef meat in Egypt. The PCR amplification results are summarized in Table 5. All except for one staphylococcal isolate (*S. lentus*) beared > two antibiotic resistant genes and a noticeable number of these staphylococci (17/27, 63.7%) carried four different antibiotic resistant genes. In particular, the nine *S. aureus* isolates carried the five antibiotic resistant genes that confer resistance to the 12 different antibiotics tested in this investigation. Seven *S. aureus*, six *S. hyicus*, four *S. schleiferi* subsp. *coagulans*, three *S. intermedius* and one *S. lentus* isolates although being resistant to oxacillin yet, 11/27 (40.7%) carried the *mec*A gene. The association between the *mec*A gene and oxacillin resistance is outlined in Table 5. At the same time, the *cfr* gene was present in 2 of the nine *S. aureus* isolates, and totally undetectable in *S. hyicus, S. schleiferi* subsp. *coagulans, S. intermedius* and *S. lentus.* At the same time, the *cfr* gene was present in two of the nine *S. aureus* isolates, and totally undetectable in *S. hyicus, S. schleiferi* subsp. *coagulans, S. intermedius*, and *S. lentus* (Table 5).

### Detailed analysis of selected isolates: species determination

The 27 isolates were chosen for further detailed examination on the basis of their ciprofloxacin and oxacillin from the disc diffusion screening. There was interspecies and intraspecies variation among the non-*S. aureus* species listed in Table 3. The four ciprofloxacin-susceptible, oxacillin -susceptible isolates examined, were *S. hyicus*. Again, four ciprofloxacin-resistant, oxacillin susceptible isolates were *S. hyicus*. Of the four ciprofloxacin-resistant, oxacillin -resistant isolates, four were *S. hyicus*, two were *S. intermedius* and *one* was *S. schleiferi* subsp. *coagulans* and *S. lentus* each. The study shows that, the occurrence of MRS from beef meat is not uncommon (Table 6). Raw beef meat sold across the Great Cairo zone, contains 66.7% of MRS (18/27), with highest prevalence was reported in *S. aureus* (6/9, 66.7%), while the MRS non-*S. aureus* strains 12/18 (66.7%) from which *S. hyicus* (6/10, 60%), *S. intermedius* (1/3, 33.3%), *S. schleiferi* subsp. *coagulans* (4/4, 100%), and *S. lentus* (1/1, 100%) were MRS.

**Table 6:** **Prevalence of *mecA* gene in beef samples (*n* = 27)**.

***Staphylococcus* spp. (*n* = isolates)**	***n* = of MRS**	**Presence of** ***mecA*** **gene**		***n* = of MSS**
		***n* =**	**%**	
*S. aureus* (*n* = 9)	6	5	55.6	3
*S. hyicus* (*n* = 10)	6	3	50	4
*S. intermedius* (*n* = 3)	1	1	100	2
*S. schleiferi* subsp. *coagulans* (*n* = 4)	4	2	50	0
*S. lentus* (*n* = 1)	1	0	0	0
Total	18	11	68.8	11

MRS, methicillin resistant Staphylococcus.

MSS, methicillin susceptible Staphylococcus.

### Survey of methicillin, oxacillin, and ciprofloxacin resistance

As seen in Table 3, the distribution of oxacillin among the non-*S. aureus* (CNS) isolates was quite broad (14/18, 77.8%). The overall frequency of ciprofloxacin resistance among non-*S. aureus* (CNS) was 44.4% (8/18), while that among methicillin-resistant non-*S. aureus* (MRCNS) was 61.1% (11/18), higher, as expected, than that for methicillin-susceptible non-*S. aureus* (MSCNS) 33.3% (6/18). The distribution of *S. aureus* and non-*S. aureus* ciprofloxacin susceptibilities as a function of methicillin susceptibilities in addition to the *mec*A resistance determinant which is responsible for the methicillin resistance, is recorded in Table 6.

## Discussion

The non-*S. aureus* species that have been isolated in the present investigation were recorded as relevant in their ability to produce human infections (*S. lentus*, Stepanovic et al., 2003; *S. hyicus*, Casanova et al., 2011; *S. schleiferi* subsp. coagulans, Davis et al., 2013; and *S. intermedius*, Koci et al., 2015).

Humans are at a risk of being infected with resistant food-borne bacteria via the food chain (Khan et al., 2000; Pesavento et al., 2007) from reservoir animals (de Jong et al., 2009; Ewers et al., 2009; O'Keefe et al., 2010; Marshall and Levy, 2011). A previous research (Waters et al., 2011) has suggested that, when buying meat at any butcher shop, supermarket or local grocery shop, is an equally likely chance of it being contaminated with drug-resistant bacteria. The WHO has revealed a report on the global serious threat of antibiotic resistant strains of bacterial pathogens occurring currently in every locality of the world with no limitations and to affect everybody, at any age and in any country (CDC, 2013).

In animals, comparison of resistance rates to antimicrobials can geographically vary among internationally and nationally according to the antimicrobial usage regime (Harada and Asai, 2010; Doyle et al., 2013) due to sample collection schemes, sample types, their minimum capability to implement AMR surveillance, rock-bottom financial and technical resources and differences in the implemented methodologies in the form of credibility, duplicability, enequitable significance, price, duration and simplicity (Strommenger et al., 2006; Cookson et al., 2007; Faria et al., 2008; Vindel et al., 2009; David et al., 2013) which makes any comparative effort to be unadvisable.

A wide dispersion of two categories of resistance has been revealed in the present investigation: 100% of our isolates carried the QRDRs responsible for quinolone resistance genes (*gyrA* and *grlA*) (17/27, 63% and 19/27, 70.4%) and the *mec*A resistance determinant (11/27, 40.7%), responsible for the methicillin and oxacillin resistance (Table 5). The *mec*A determinant has caused severe complications during the treatment MRSA in humans (Lee, 2003) and for *S. epidermidis* infections too (Kitao, 2003) when the hypothesis that, *mec*A is transferred from non-*S. aureus* staphylococci to *S. aureus* (Grundmann et al., 2006; Bhargava and Zhang, 2014) is taken into consideration.

The French National Monitoring Program (RESABO) established evidence that *S. aureus* is more susceptible to antimicrobial drugs than non-*S. aureus* species (Werckenthin et al., 2001). Conversely, our results had a commensurate regular and sequence discernible in the antibiotic resistance genes between *S. aureus* and non-*S. aureus* isolates. The results showed a congruity between phenotypic resistance and molecular genotyping for oxacillin, QRDRs, and PhLOPSA phenotypes. Generally, our results are in assonance with Chajęcka-Wierzchowska et al. (2015) who displayed that, all methicillin resistant staphylococci harbored *mec*A gene but in consonance with the investigation outcomes of Martineau et al. (2000), Lee et al. (2004), and Guran and Kahya (2015), who indicated the occurrence of staphylococci strains resistant to methicillin or/and oxacillin in the absence of the *mec*A gene. The reason behind this variable expression could be related to the hyperproduction of β-lactamase type A (Martineau et al., 2000) and/or cellular growth conditions according to Lee et al. (2004). Globally, there are considerable discrepancies between different countries in the MRSA prevalence rate on beef meat (Normanno et al., 2007; van Loo et al., 2007a,b; Chan et al., 2008; Pu et al., 2009; Lim et al., 2010; Weese, 2010; Weese et al., 2010; Bhargava et al., 2011; Chua et al., 2011; Hanson et al., 2011; Dalhoff, 2012; O'Brien et al., 2012; Buyukcangaz et al., 2013; Jackson et al., 2013; Nnachi et al., 2014; WHO, 2014).

The animals are the first reservoirs for CNS bearing the *cfr* resistance gene which endows resistance to phenicols, lincosamides, oxazolidinones, pleuromutilins, and streptogramin A antibiotics (PhLOPS_A_ of phenotype) and some macrolides (Kehrenberg et al., 2005). An interesting and fortunate observation was that, we detected the gene *cfr* in 22.2% (2/9) of the *S. aureus* isolates, a rate which was lower than the detection rates of food animal samples in previous studies (Wang et al., 2012; Zeng et al., 2014) while the non-*S. aureus* isolates did not carry this gene. The c*fr* multiresistance gene is usually found in isolates of human origin (Chen et al., 2013; Cui et al., 2013) and persistence in retail meat (LaMarre et al., 2011). The insignificant detection rate found in this study (2/27, 7.4%), indicated the neglible opportunity of the *cfr* to be spread through contaminated meat with *Staphylococci* in the local market by consumers in Egypt, the diminished possibility of this gene to enter the food cycle, and the chance of additional distribution of *cfr* from an animal reservoir to *S. aureus*, MRSA or any other staphylococcal species associated with colonization and infection in humans (Witte and Cuny, 2011) is abolished. Timely detection and targeted prevention of further dissemination of MRSA present on retail beef meat and the *cfr* gene becomes justified in order to preserve efficacy of linezolid due to its importance and preference in the treatment of staphylococcal infections (Witte and Cuny, 2011).

Fluoroquinolones (FQs) are antibiotics effective against a wide range of organisms (Oliphant and Green, 2002) and having a role in the chemotherapy and postexposure prophylaxis for organisms, that could be used in biologic warfare. The extensive use of FQs has led to the springing up of FQ-resistant *S. aureus*, especially among MRSA strains (Oliphant and Green, 2002) in the QRDRs of the *gyr*A, *gyr*B, *grl*A, and *grl*B genes (Hooper, 2002; Oliphant and Green, 2002; Redgrave et al., 2014; Parkinson et al., 2015). Gade and Qazi (2013) signified that, ciprofloxacin can no longer be used as an empirical therapy against MRSA infections to be replaced by vancomycin as the alternative drug to which we are in dispute as we recorded a 77.8% resistance in the MRSA. It should be noted that, the access to any available literature on any information concerning the *gyr*A, *gyr*B, *grl*A, and *grl*B genes in meat was not available.

The contamination of carcasses with MRSA can occur via several means: (i) improper agricultural practices, (ii) use of impotable water for agricultural irrigation, (iii) the use of improperly disinfected appliancies used in the food industry, (iv) unsuitable temperatures during storage and transportation, (v) poor food handling procedures, vi) lack of rodent and/or insect control (FDA, 2012; WHO, 2012), (vii) through the butchers tools which are suitable vehicles for transmission (de Boer et al., 2009), (viii) on the meat display table, to buyers and vice versa (Emele et al., 1995). The environmental contamination of the slaughter-house, processing appliances or operative hands in these facilities with MRSA could result in a cross-contagion process to the meat (Wertheim et al., 2004; Alexandra et al., 2011), and consequently conceal the importance of the association between on-farm antibiotic use and contamination of the meat with MRSA. The most important outcome of our investigation was the alarming finding that, most of the MRSA and NSA-MR isolated from retail meat in the Egyptian market were >50% resistant to clinically important antimicrobials (sulfamethoxazole/trimethoprim, clindamycin, gentamycin, peniciilin, oxacillin, vancomycin, tetracycline, methicillin, ciprofloxacin, and erythromycin in descending order) imposing a severe threat to public health of the Egyptians. In addition, the prevalence rate of the MRSA and NSA-MR isolated from retail meat collected from sale outlets reflected an indication to the level of exposure of the consumers to these pathogens (Wagenaar et al., 2007).

## Conclusions

To the best of our knowledge, the extent of a similar research topic has not been tackled in Egypt. In addition, the strength of our study lies in the fact that, previous researches on *S. aureus* isolated from foodstuffs were merely screened for the *mec*A gene, while in our investigation a selective enrichment of MRSA was adopted to be followed by a PCR technique for the identification for the presence of *mec*A gene. Also, we included in our investigation samples collected from three different geographic areas involving both municipal and agricultural sectors. In some of our strains, we found that although resistance was recorded, yet the responsible gene was not expressed which could be due to, the small number of resistance genes tested or/and the gene responsible for resistance was not included in our protocol. There are no existing programs for surveillance which allows us to recommend a large scale of research to be conducted to cover the whole country with specific zone system, specifying large number of sample size to be included covering most type of meat consumed in Egypt.

## Author contributions

KO developed the concept, designed experiments, collected, and analyzed data and prepared the manuscript; AA provided reagents and mice; JB and NH performed PCR assays; RE gave technical support and conceptual advice; AO performed PCR assays; MB gave technical support and conceptual advice; AS performed the experiments. All authors discussed the results and implications and commented on the manuscript at all stages.

### Conflict of interest statement

The authors declare that the research was conducted in the absence of any commercial or financial relationships that could be construed as a potential conflict of interest.
